# IgG4-Related Orbital Disease vs. Idiopathic Orbital Inflammation: Clinical Features, Therapy and Outcomes in a Central-European Retrospective Single-Center Cohort

**DOI:** 10.3390/biomedicines13092311

**Published:** 2025-09-21

**Authors:** Alexander Lukas Rattunde, Vitus André Knecht, Eckart Bertelmann

**Affiliations:** Department of Ophthalmology, Charité–Universitätsmedizin Berlin, Corporate Member of Freie Universität Berlin and Humboldt-Universität zu Berlin, Augustenburger Platz 1, 13353 Berlin, Germany

**Keywords:** IgG4-Related Orbital Disease (IgG4-ROD), Idiopathic Orbital Inflammatory Syndrome (IOIS), orbital inflammation, differential diagnosis, immunosuppressive therapy

## Abstract

**Objective:** IgG4-Related Orbital Disease (IgG4-ROD) is an incompletely understood differential of idiopathic orbital inflammatory syndrome (IOIS). Accurate separation guides therapy and prognosis. This retrospective study also profiles its clinical features, therapy needs, and compares them with IOIS. **Methodology:** Fifty-four patients were biopsied between January 2016 and December 2023, comprising 18 who were diagnosed with IgG4-ROD (4 definite, 14 probable) and 36 with IOIS. Mean follow-up was 21.7 ± 26.2 months for IgG4-ROD versus 7.5 ± 10.3 months for IOIS. **Results:** Patients with IgG4-ROD were older than those with IOIS (mean 61.8 vs. 49.9 years). Gender distribution was balanced. The lacrimal gland (66.7% vs. 61.6%; *p* = 0.690) and extra-ocular muscles (55.6% vs. 30.6%; *p* = 0.076) were frequently involved in both entities, whereas paranasal sinus infiltration was significantly associated with IgG4-ROD (*p* = 0.003). Common shared symptoms (*p* > 0.05) included eyelid swelling (83.3% vs. 86.1%), exophthalmos (50% vs. 36.1%), and motility restriction (22.2% vs. 25%). Relative afferent pupillary defect (*p* = 0.042), chemosis (*p* = 0.02), and systemic disease (*p* = 0.005) were more prevalent in IgG4-ROD. During ≥ 6-month follow-up (*n* = 7), only 28.6% of IgG4-ROD patients achieved sustained remission; Kaplan–Meier analysis yielded a mean time to first event of 926 days. Additional steroid-sparing therapy was required more often in IgG4-ROD than in IOIS (*p* = 0.002). **Conclusion:** IgG4-ROD and IOIS share clinical features but differ in key aspects such as associated diseases, therapy requirements, and disease control. Understanding these differences is crucial for targeted diagnostics and individualized treatment strategies.

## 1. Introduction

Inflammatory orbital diseases (IODs) include myositis, dacryoadenitis, and diffuse inflammation that can involve the lacrimal gland, extraocular muscles, and other orbital soft tissues [1,2,3]. Histology typically shows a mixed lymphoplasmacytic infiltrate with variable fibrosis and no specific immunohistochemical marker [4]. Clinically, presentations range from eyelid swelling and proptosis to optic nerve compromise. IODs are the third most common orbital disease after thyroid eye disease and orbital lymphoma [2,5,6,7,8]. By definition, idiopathic orbital inflammatory syndrome (IOIS) is a diagnosis of exclusion, and secondary systemic causes preclude IOIS [9].

IgG4-related disease (IgG4-RD) is a multisystem fibro-inflammatory condition that forms mass-like lesions in many organs, including the orbit as IgG4-related orbital disease (IgG4-ROD) [10,11,12]. IgG4-ROD often presents with (bilateral) lacrimal gland enlargement and may involve branches of the trigeminal nerve and the extraocular muscles [13,14]. Diagnostic criteria have evolved over time. The 2015 ophthalmic criteria combined imaging, histopathology, and serology (>135 mg/dL) [14]. The 2023 updates place particular emphasis on optic neuropathy and broaden the lymphoma differential beyond mucosa-associated lymphoid tissue (MALT) to include other subtypes such as diffuse large B-cell lymphoma (DLBCL). Under these updates, certainty is graded as definite, probable, or possible based on combinations of imaging, histology, and serology [15,16,17,18,19]. Across IgG4-RD, including IgG4-ROD, histopathology shows dense lymphoplasmacytic infiltrates, storiform fibrosis, and obliterative phlebitis with increased IgG4-positive plasma cells [20,21].

IgG4-ROD is common within cohorts previously labeled IOIS. Reported proportions range from about 24% to 60%, and a French series found 37% [17,22,23,24]. Within the sclerosing subtype of IOIS, which carries a worse prognosis, up to 46% met criteria for IgG4-ROD [6,25]. Current evidence supports an immune-driven mechanism that involves CD4+ T cells and various cytokines, which promote class switching, plasma-cell survival, and fibrosis [11,26]. Compared with IOIS, IgG4-ROD more often recurs, more frequently involves the optic nerve, and often requires steroid-sparing immunosuppression [11,15,27,28,29]. Because clinical signs overlap, including eyelid swelling, proptosis, and lacrimal gland enlargement, biopsy remains central to differentiation [6,28].

Most comparative data come from Asian cohorts, especially from China and Japan [30]. To address the regional gap, we analyze a Central-European, biopsy-based cohort and directly compare clinical features, therapy, and outcomes in IgG4-ROD and IOIS.

## 2. Materials and Methods

This retrospective, single-center study included 54 patients who underwent orbital biopsy between January 2016 and December 2023. We analyzed a total of 18 patients who were subsequently diagnosed with IgG4-ROD and 36 patients with IOIS. We classified every study participant according to the 2023 revised diagnostic criteria for IgG4-related orbital disease (IgG4-ROD) formulated by Takahira and colleagues [15]. The algorithm recognizes three mutually exclusive categories:

Definite IgG4-ROD: (1) characteristic orbital imaging (e.g., lacrimal gland, trigeminal nerve, or extra-ocular-muscle enlargement or focal hypertrophic masses) plus (2) histopathology showing dense lymphoplasmacytic infiltration with either an IgG4^+^/IgG^+^ cell ratio ≥ 40% or > 50 IgG4^+^ plasma cells per high-power field (HPF), plus (3) elevated serum IgG4 >135 mg/dL (1.35 g/L).

Probable IgG4-ROD: criteria (1) and (2) are satisfied, but serum IgG4 is normal or unavailable. Possible IgG4-ROD: criteria (1) and (3) are satisfied in the absence of histology.

Orbital MRI reports were reviewed by two fellowship-trained neuro-radiologists, and all biopsy specimens were reassessed by board-certified ophthalmic pathologists who applied the quantitative IgG4 immunostaining cut-offs above. Serum IgG4 was measured with a nephelometric assay; the laboratory upper limit of normal is 1.35 g/L. Of our 18 IgG4-ROD patients, all patients were biopsy-proven and fulfilled clinical and/or radiological criteria, whereas 4 patients also met the serologic threshold. Given that serology lacks perfect sensitivity and specificity, characteristic, biopsy-proven histopathology is decisive for IgG4-ROD. Furthermore, treatment does not differ by serologic status. Therefore, we analyzed definite and probable cases together as one clinical entity.

Patients were followed until August 2023 or until a follow-up loss.

Of the 54 orbital-biopsy cases screened for this study, the remaining 36 (66.7%) satisfied our working definition of idiopathic orbital inflammatory syndrome and comprise the IOIS comparison cohort. Each lesion was biopsy-proven: histology showed a polymorphous lymphoplasmacytic infiltrate without granuloma, vasculitis, necrosis, or malignancy, and IgG4 immunostaining remained below the diagnostic thresholds for IgG4-RD (≤30 IgG4-positive plasma cells per high-power field and an IgG4^+^/IgG^+^ ratio < 40%). Baseline serum IgG4 concentrations, when available, were consistently below the laboratory upper limit of normal (1.35 g/L), further excluding IgG4-related disease. These 36 patients, therefore, constitute the IOIS group used for all subsequent analyses reported alongside the IgG4-ROD cohort.

Formal inclusion criteria were biopsy-proven orbital inflammation/mass seen at our center between January 2016 and December 2023, with baseline clinic documentation and an orbital MRI report. Group assignment followed the 2023 ophthalmic criteria: IgG4-ROD required compatible imaging plus lymphoplasmacytic inflammation on biopsy with quantitative IgG4 immunostaining (categorized as definite or probable as defined above); IOIS required a polymorphous lymphoplasmacytic infiltrate on biopsy without granuloma/vasculitis/necrosis/malignancy and below the IgG4 thresholds as specified earlier. Exclusion criteria were infection, neoplasm (including lymphoma), thyroid eye disease, granulomatous or vasculitic disorders, insufficient tissue for histology/IgG4 assessment, or incomplete core clinical/radiologic documentation precluding classification.

All study variables were obtained exclusively by retrospective review of the electronic health record; no additional examinations, laboratory tests, or imaging were ordered for research purposes. Demographic data (age, sex) and ophthalmic findings such as eyelid status, pupillary reactions (including documentation of any relative afferent pupillary defect), ocular motility, and best-corrected visual acuity before and after treatment were abstracted from routine clinic notes. Anatomical involvement of the lacrimal gland, extra-ocular muscles, paranasal sinuses, and other orbital tissues was recorded from existing MRI reports. Histopathology reports were examined for confirmation of IgG4-positive plasma cells and for qualitative comments on the degree of inflammation and fibrosis; quantitative cell densities were not consistently documented and were therefore not analyzed. Finally, systemic manifestations (e.g., pancreatitis, parotitis, sinusitis, orchitis, arthralgias) and all pharmacological treatments initiated by rheumatologists or ophthalmologists, including first-line corticosteroids and any subsequent immunosuppressive agents such as azathioprine, cyclophosphamide, or rituximab, were extracted from the clinical documentation.

To ensure consistent, time-anchored assessment of treatment efficacy across all charts, we applied a uniform set of outcome definitions that were coded prospectively at every follow-up visit (Table 1):

Index date (T_0_)—start of systemic corticosteroid therapy. Initial response (IR), first visit ≤ 6 months after T_0_ with complete absence of symptoms and no active ocular-exam signs.

Remission, any visit ≥ 6 months after T_0_ with (a) no symptoms and (b) no active signs. Loss of disease control, recurrence of symptoms or signs prompting escalation of therapy after a documented initial response.

The coding rules were set a priori and used verbatim during data abstraction. A complete-case analysis was performed. Because IOIS patients were frequently followed outside our tertiary center without standardized ≥ 6-month documentation, outcome analyses (IR, remission, loss of disease control) were prespecified for the IgG4-ROD cohort only.

### Statistical Analysis

All analyses were carried out in GraphPad Prism, version 10.3.1 (GraphPad Software, San Diego, CA, USA). Continuous variables were inspected for normality with the Shapiro–Wilk test. Depending on the result, they are reported as mean ± standard deviation and compared with the unpaired Student *t*-test, or as median (inter-quartile range) and compared with the two-tailed Mann–Whitney U test. Categorical data are expressed as counts and percentages and were examined with the Chi^2^ test; Fisher’s exact test was substituted when an expected cell size was < 5.

For the treatment-outcome end points, initial response (IR), remission, and loss of disease control, simple frequencies were calculated. Time to loss of disease control was summarized with Kaplan–Meier survival analysis; patients who had not experienced an event at their last documented visit were censored on that date. Median relapse-free survival and 95% Hall–Wellner confidence bands are reported.

Exclusion criteria for each analysis were pre-specified. Patients with < 6 months of follow-up were excluded from the outcome analyses but remained eligible for all baseline descriptive comparisons. Variables with missing values were handled by complete-case analysis; the denominator (*n*) is stated for every result. All tests were two-sided, and *p* < 0.05 was considered statistically significant.

## 3. Results

### 3.1. Patient Demographics

Eighteen consecutive patients fulfilled the clinical entry criteria for IgG4-ROD. All 18 met both the imaging criterion and the histopathology criterion, whereas baseline serum IgG4 was available in 14 cases (78%). The median value of serum IgG4 concentration was 0.81 g/L (inter-quartile range 0.46–1.08; full range 0.22–5.40 g/L). Four patients (22%) had levels > 1.35 g/L. Four out of eighteen patients (22%) satisfied all three criteria and were thus classified as definite IgG4-ROD. The remaining 14 patients (78%) fulfilled imaging and histology criteria but either had a normal serum IgG4 (*n* = 10) or the assay was not performed (*n* = 4). They were consequently labeled as probable IgG4-ROD.

Thus, every patient in our cohort was classified as either probable or definite IgG4-ROD; no case met the “possible” category because histological confirmation was universal.

The mean follow-up duration was 21.72 months (SD ± 26.16) in the IgG4-ROD group and 7.5 months (SD ± 10.33) in the IOIS group (Table 2). Patients with IgG4-ROD were significantly older compared to those with IOIS (61.78 ± 15.85 years vs. 49.94 ± 15.04 years, *p* = 0.02). The sex distribution was balanced in both groups (*p* = 0.564).

### 3.2. Systemic and Orbital Involvement

**Figure 1:**
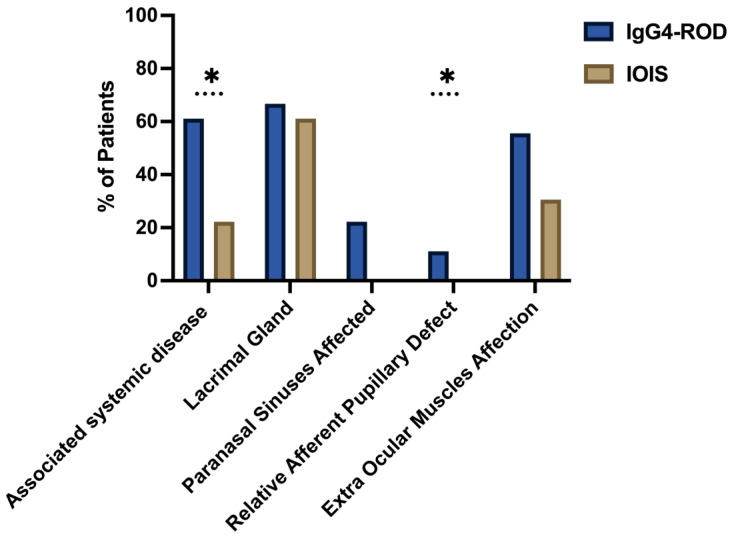
Comparison of clinical features of IgG4-ROD = IgG4-Related Orbital Inflammatory Syndrome and IOIS = Idiopathic Orbital Inflammatory Syndrome. The figure depicts a comparative analysis of the clinical characteristics between IgG4-ROD and idiopathic orbital inflammatory syndrome (IOIS), in relation to the percentage of affected patients. The blue color represents cases of IgG4-ROD, while the brown color represents IOIS. The percentage of patients affected is indicated on the y-axis. Statistically significant changes are marked with an asterisk (*).

Systemic disease associations (e.g., pancreatitis, parotitis, sinusitis, orchitis, and arthralgias) were documented in 61.1% of IgG4-ROD patients and 22.2% of IOIS patients (*p* = 0.005). The percentages of patients in each group who presented with systemic manifestations are illustrated in Figure 1.

Regarding orbital structures (Figure 1), lacrimal gland involvement was observed in 66.7% of patients with IgG4-ROD and 61.1% of patients with IOIS (*p* = 0.69). Extraocular muscle infiltration occurred more frequently in IgG4-ROD (55.6%) compared to IOIS (30.6%) (*p* = 0.076). Paranasal sinus involvement was significantly higher in IgG4-ROD (22.2%) compared with IOIS (0%) (*p* = 0.003).

Frequent clinical symptoms included eyelid swelling, exophthalmos, and motility restriction in both groups. Chemosis was observed in 22.2% (IgG4-ROD) and 2.8% (IOIS) (*p* = 0.02). A relative afferent pupillary defect (RAPD) was detected in 11.1% (IgG4-ROD) vs. 0% (IOIS) (*p* = 0.042).

### 3.3. Visual Acuity

**Figure 2:**
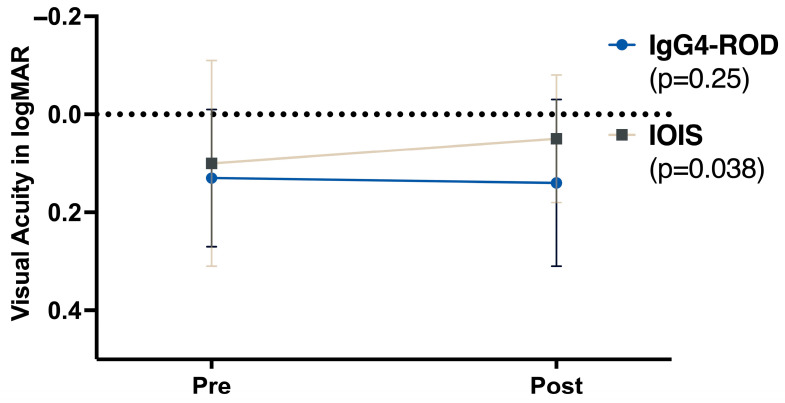
Visual acuity (logMAR) before and after treatment. Mean logMAR values at baseline (Pre) and after therapy (Post) for IgG4-ROD (blue markers) and IOIS (brown markers). A lower logMAR value indicates better visual acuity. IgG4-ROD = immunoglobulin G4-related orbital disease, IOIS = idiopathic orbital inflammatory syndrome.

Mean logMAR at baseline was 0.133 in IgG4-ROD (*n* = 17) and 0.102 in IOIS (*n* = 35; *p* = 0.58). At the last visit, means were 0.131 (*n* = 11) and 0.050 (*n* = 26). Pre/post analyses were restricted to paired eyes only. A significant improvement in best-corrected visual acuity (BCVA) after treatment was documented in the IOIS group (*p* < 0.05). In contrast, no statistically significant change was noted in BCVA in the IgG4-ROD group (*p* = 0.25). Figure 2 summarizes pre- and post-treatment BCVA measurements.

### 3.4. Therapy and Outcomes

**Figure 3:**
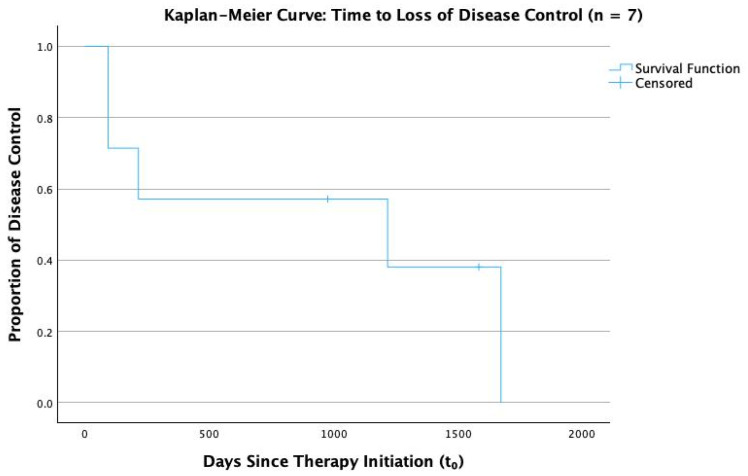
Kaplan–Meier curve: Time to Loss of Disease Control.

Seven IgG4-ROD patients were eligible for outcome analysis. Four (57.1%) achieved an initial response within 6 months. At the last follow-up, two (28.6%) remained in remission, whereas five (71.4%) lost disease control and required treatment escalation (Figure 3).

Systemic therapy lines are summarized in Table 3. In IgG4-ROD, 10/15 treated patients escalated beyond steroids (second-line 7/18, multi-agent 3/18). In IOIS, most received no systemic therapy or steroids only (35/36), and systemic escalation was rare 1/36).

Therapeutic modalities are shown in Table 4: IgG4-ROD frequently used cytotoxic immunosuppressants (azathioprine 7/18, cyclophosphamide 3/18, methotrexate 1/18) and rituximab 4/18 (1–2 × 1000 mg per cycle; 2–4 cycles total), whereas IOIS more often received radiotherapy (6/36) with minimal exposure to cytotoxic agents (methotrexate 1/36).

## 4. Discussion

Despite the small sample sizes that frequently pertain to inflammatory orbital diseases, our study was able to demonstrate that while IgG4-ROD and IOIS share certain clinical features, they differ in key findings and prognostic measures.

In our cohort, patients with IgG4-ROD were, on average, older than those with IOIS. The mean age of 61.8 years is in line with what has been reported by other authors [31,32]. Paranasal sinus involvement was significantly more frequent in patients with IgG4-ROD, which concurs with evidence hinting at sinus involvement in IgG4-RD [22,33,34] and thus is an important distinguishing feature.

While our data suggest a trend toward more frequent bilateral orbital involvement in patients with IgG4-ROD, we were unable to achieve statistical significance (*p* = 0.066), as reported also by other studies [35,36,37]. This limitation is likely attributable to insufficient statistical power in our analysis.

In patients presenting with gradual development of unilateral proptosis of unknown origin, there is rarely conclusive evidence of either neoplastic or inflammatory origin prior to imaging and biopsy, much less the subgroup of possible inflammatory etiology. Since IgG4-ROD is inherently a manifestation of a systemic rheumatologic condition, it is essential to obtain a comprehensive patient history. This should include a detailed account of any prior rheumatologic diagnoses as well as an assessment for symptoms indicative of systemic involvement. Our data show that in patients later diagnosed with either IgG4-ROD or IOIS, there is a statistically significantly higher prevalence of such symptoms in patients with IgG4-ROD on initial presentation, notably pancreatitis, orchitis, parotitis, and sinusitis. Recognizing and addressing systemic disease manifestations remains vital for comprehensive care. In IgG4-RD, the involvement of other organ systems, such as the kidneys, lungs, and pancreas, may severely impact morbidity or even mortality. Serious complications due to periaortitis, severe retroperitoneal fibrosis, or pachymeningitis may arise [11], necessitating multidisciplinary management. In our cohort, 14/18 IgG4-ROD cases were categorized as probable because serum IgG4 was normal despite characteristic imaging and biopsy. This seronegative presentation is well documented: serum IgG4 has incomplete sensitivity and limited specificity, and a meaningful subset of biopsy-proven IgG4-RD have normal levels; hence, histopathology remains the diagnostic anchor [38,39]. Because treatment does not differ by serostatus, we analyzed definite and probable together while stating diagnostic certainty explicitly. Additionally, there is a growing body of evidence suggesting increased incidence of orbital lymphoma in patients with IgG4-ROD [16,17,18], further demonstrating the importance of early recognition and possibly the need for long-term follow-up.

Furthermore, the data showed a higher frequency of asthma and atopic diathesis in IgG4-RD patients, which is in line with prior findings associating the two conditions [40,41,42].

Optic neuropathy remains a serious complication of all inflammatory orbital mass lesions. In our study, patients with IgG4-ROD experienced a higher frequency of relative afferent pupillary defect (RAPD), suggesting a higher frequency of severe orbital affection. This aligns with updated 2023 diagnostic criteria for identifying severe cases of IgG4-ROD with optic nerve involvement [15].

In addition to optic neuropathy, 71% of evaluable IgG4-ROD patients lost disease control within 4.6 years. This is in accordance with evidence suggesting that cases of IgG4-ROD and the sclerosing subtype of IOIS often retrospectively reclassified as IgG4-ROD have historically demonstrated a severe and treatment-resistant course [25,27,43].

Qualitatively, treatment trajectories diverged: most IgG4-ROD patients required step-up, multi-agent immunosuppression (e.g., rituximab, azathioprine, cyclophosphamide) after an initial corticosteroid course, whereas IOIS cases were generally controlled with steroids alone or even without any treatment. This pattern underscores the need for long-term monitoring and an interdisciplinary approach when managing IgG4-ROD.

It is therefore important for clinicians to remain vigilant for IgG4-ROD, particularly when patients exhibit recalcitrant disease refractory to steroids, those with severe orbital affection, or in cases of bilateral orbital inflammatory disease. Early multidisciplinary involvement to monitor for systemic disease is crucial. Failure to recognize IgG4-ROD as such in a timely manner may increase ophthalmic and systemic morbidity.

Future research should focus on comparing the radiological features of IgG4-ROD with IOIS. Although common radiological characteristics of IgG4-ROD, such as infraorbital nerve enlargement, sinus involvement, bilateral disease, and sparing of muscle tendons, have been described in previous studies [37,44], it would be particularly valuable to directly contrast these findings with those of IOIS or even malignant orbital lesions. Such comparative analyses, when combined with clinical features, radiological presentations, and systemic manifestations, could help improve pre-biopsy diagnostic accuracy in distinguishing IgG4-ROD from other orbital pathologies.

Another critical area of future investigation is the determination of individualized treatment strategies for IgG4-ROD. Prospective studies with long-term follow-up are essential to evaluate the efficacy and safety of different therapeutic approaches. Establishing a national or European database for IgG4-ROD cases would also significantly enhance the ability to conduct meaningful research. Such a database could address the challenges posed by the rarity of the disease, enabling more robust comparisons and better-informed treatment regimens through larger sample sizes and pooled data.

## 5. Limitations

This study is a retrospective analysis from a single tertiary referral center; thus, the limitations pertaining to retrospective design apply to our study. As such, some data elements (e.g., paired initial and follow-up visual acuity, standardized symptom reporting, and other follow-up details) were inconsistently documented in the charts, so certain analyses could only be reported descriptively or were not feasible; an inherent constraint of retrospective reviews. Follow-up duration differed between the IgG4-ROD and IOIS groups, which meant formal time-to-event comparisons were not feasible, and survival estimates were restricted to the IgG4-ROD cohort. This may also have affected the likelihood of patients in either cohort requiring second-line therapy. Imaging protocols, histopathological reporting, and treatment regimens reflected everyday clinical practice rather than a prespecified research pathway, so quantitative counts of IgG4-positive plasma cells were not always recorded, and systemic therapies varied in agent, dose, and duration; similarly, four patients lacked baseline serum IgG4 levels, and those missing data were handled by complete-case analysis. These factors inevitably widen confidence intervals and limit definitive statements about the relative effectiveness of individual immunosuppressive agents. Even so, the dataset offers a valuable real-world snapshot of IgG4-ROD within a referral setting and underscores the frequent need for prolonged, multi-agent therapy. Prospective, multicenter studies that employ standardized diagnostic criteria and treatment algorithms will be essential to confirm and extend these observations.

## Figures and Tables

**Table 1:** Outcome definitions.

Variable	Coding Rule (Applied to Every Follow-Up Note)
Index date T_0_	Start of systemic steroids (or biopsy date if untreated)
Initial response (IR)	First visit ≤ 6 months with no symptoms and no active exam signs
Remission	Any visit ≥ 6 months with (a) no symptoms and (b) no active signs
Loss of disease control	Recurrence of symptoms/signs requiring treatment escalation after an IR

**Table 2:** Demographics and clinical characteristics.

Parameter	IgG4-ROD	IOIS	*p*-Value	Significant Difference
Patient Demographics				
Average Age (years)	61.78 ± 15.85	49.94 ± 15.04	0.02	*
Female/Male Patients (%)	44.44%/55.56%	52.78%/47.22%	0.564	
Mean follow-up duration (months)	21.72 ± 26.16	7.5 ± 10.33	0.02	*
Associated systemic disease (%)	61.11%	22.22%	0.005	*
Lacrimal Gland Involvement (%)	66.67%	61.11%	0.690	
Extraocular Muscle Involvement (%)	55.56%	30.56%	0.076	
Paranasal Sinus Involvement (%)	22.22%	0%	0.003	*
Clinical Symptoms				
Eyelid Swelling (%)	83.33%	86.11%	0.786	
Exophthalmos (%)	50.00%	36.11%	0.327	
RAPD (%)	11.11%	0.00%	0.042	*
Chemosis (%)	22.2%	2.8%	0.02	*

This table represents the demographics and clinical characteristics of all patients, the IgG4-ROD group and the IOIS group. The *p*-value represents the significance of the differences between the two groups. RAPD = Relative Afferent Pupillary Defect, IgG4-ROD = immunoglobulin G4-related orbital disease, IOIS = idiopathic orbital inflammatory syndrome. * Statistically significant, *p* < 0.05 (two-tailed).

**Table 3:** Systemic therapy lines by cohort.

Systemic Therapy Line	IgG4-ROD (*n* = 18)	IOIS (*n* = 36)
No systemic therapy (incl. radiotherapy-only)	3 (16.7%)	11 (30.6%)
First-line only: steroids (±local therapy)	5 (27.8%)	24 (66.7%)
Escalated: ≥ 1 steroid-sparing systemic agent	7 (38.9%)	1 (2.8%)
Multi-agent escalation: ≥ 2 steroid-sparing agents	3 (16.7%)	0 (0.0%)

IOIS notes: “No systemic” = 10 with no treatment + 1 with radiotherapy alone. “First-line only” = 19 steroids alone + 4 steroids+RT + 1 steroids+RT+exenteration. IgG4-ROD = immunoglobulin G4-related orbital disease, IOIS = idiopathic orbital inflammatory syndrome.

**Table 4:** Therapies and modalities used (patients exposed at any time).

Modality	IgG4-ROD (*n* = 18)	IOIS (*n* = 36)
Cytotoxic immunosuppressants (any)	9 (50.0%)	1 (2.8%)
Azathioprine	7 (38.9%)	0 (0.0%)
Cyclophosphamide	3 (16.7%)	0 (0.0%)
Methotrexate	1 (5.6%)	1 (2.8%)
Rituximab †	4 (22.2%)	0 (0.0%)
Radiotherapy	2 (11.1%)	6 (16.7%)
Exenteration	0 (0.0%)	1 (2.8%)

† Rituximab dosing: 1 × 1000 mg or 2 × 1000 mg per cycle; 2–4 total cycles. IgG4-ROD = immunoglobulin G4-related orbital disease, IOIS = idiopathic orbital inflammatory syndrome.

## Data Availability

Dataset available on request from the authors.

## Abbreviations

The following abbreviations are used in this manuscript:

BCVA	Best-corrected visual acuity
DLBCL	Diffuse large B-cell lymphoma
HPF	High-power field
IgG4-ROD	IgG4-related orbital disease
IgG4-RD	IgG4-related disease
IOD	Inflammatory orbital disease
IOIS	Idiopathic orbital inflammatory syndrome
logMAR	Logarithm of the minimum angle of resolution
MRI	Magnetic resonance imaging
MALT	Mucosa-associated lymphoid tissue
RAPD	Relative afferent pupillary defect
SD	Standard deviation
TED	Thyroid eye disease
